# Metabolomic and Proteomic Profiles Associated With Ketosis in Dairy Cows

**DOI:** 10.3389/fgene.2020.551587

**Published:** 2020-12-16

**Authors:** Zhou-Lin Wu, Shi-Yi Chen, Shenqiang Hu, Xianbo Jia, Jie Wang, Song-Jia Lai

**Affiliations:** Farm Animal Genetic Resources Exploration and Innovation Key Laboratory of Sichuan Province, Sichuan Agricultural University, Chengdu, China

**Keywords:** dairy cow, clinical ketosis, serum, metabolomics, proteomics, integrated pathway analysis

## Abstract

Ketosis is a common metabolic disease in dairy cows during early lactation. However, information about the metabolomic and proteomic profiles associated with the incidence and progression of ketosis is still limited. In this study, an integrated metabolomics and proteomics approach was performed on blood serum sampled from cows diagnosed with clinical ketosis (case, ≥ 2.60 mmol/L plasma β-hydroxybutyrate; BHBA) and healthy controls (control, < 1.0 mmol/L BHBA). Samples were taken 2 weeks before parturition and 2 weeks after parturition from 19 animals (nine cases, 10 controls). All serum samples (*n* = 38) were subjected to Liquid Chromatography-Mass Spectrometry (LC-MS) based metabolomic analysis, and 20 samples underwent Data-Independent Acquisition (DIA) LC-MS based proteomic analysis. A total of 97 metabolites and 540 proteins were successfully identified, and multivariate analysis revealed significant differences in both metabolomic and proteomic profiles between cases and controls. We investigated clinical ketosis-associated metabolomic and proteomic changes using statistical analyses. Correlation analysis of statistically significant metabolites and proteins showed 78 strong correlations (correlation coefficient, *R* ≥ 0.7) between 38 metabolites and 25 proteins, which were then mapped to pathways using IMPaLA. Results showed that ketosis altered a wide range of metabolic pathways, such as metabolism, metabolism of proteins, gene expression and post-translational protein modification, vitamin metabolism, signaling, and disease related pathways. Findings presented here are relevant for identifying molecular targets for ketosis and biomarkers for ketosis detection during the transition period.

## Introduction

Dairy cows undergo both dramatic physiological and metabolic changes during the transition period, during which a metabolic disorder known as negative energy balance commonly occurs because the actual feed intake cannot meet the largely increased nutrient requirement for milk production (Esposito et al., 2014). To compensate for the negative impact of negative energy balance, a series of physiological adaptations, including enhanced fat mobilization, hepatic gluconeogenesis, and bone resorption, were adopted to produce more energy (Reynolds et al., 2003; Itle et al., 2015). Adipose tissue metabolism is an essential contributor to successful lactation (Khan et al., 2013), but massive fat mobilization accelerates non-esterified fatty acids (NEFA) concentration in the blood. NEFA can be either uptaken by the mammary gland for milk fat synthesis or utilized by the liver for energy production (Sun et al., 2016). Because the ruminant liver has a limited capacity to completely oxidize NEFAs and synthesize very-low-density lipoprotein, excessive fatty acids will be then metabolized into ketones or esterification to form triglycerides (Bezerra et al., 2014; White, 2015) and excess ketone accumulation that can ultimately lead to ketosis (Duffield et al., 2009). In practice, ketosis is one of the most common metabolic diseases in dairy herds, with the prevalence ranging from 6.9 to 43% (Duffield, 2000; McArt et al., 2012; Suthar et al., 2013). Importantly, ketosis has a severe effect on the production performance and increases the risk of developing displaced abomasum, lameness, and metritis (McArt et al., 2012; Raboisson et al., 2014). In addition, cows with ketosis could succumb to insulin resistance, oxidative stress (Youssef and El-Ashker, 2017), hepatic apoptosis, and oxidative and inflammatory response (Du et al., 2018). However, the mechanism of the incidence and progression of ketosis is not completely understood.

Metabolomic technique is a powerful tool for elucidating disease etiologies and identifying biomarkers for disease diagnosis, detection, and monitoring (Xia et al., 2013), and it is also helpful to dissect the complex biological mechanisms of ruminants (Sun et al., 2017; Guo et al., 2019). Recently, it has been used to explore metabolic alterations and identify the predictive and diagnostic biomarkers related to clinical mastitis (Dervishi et al., 2017). Notably, metabolomics have become an attractive analytical tool with high accurate predictive, diagnostic, and prognostic abilities in studies related to cow ketosis (Zhang et al., 2017). Nevertheless, metabolites involved in ketosis are not completely clear, and effective predictive biomarkers for ketosis are still lacking. Proteome represents the summative effects of gene function and has emerged as an important tool to explore complex biological processes. For example, a recent study addressed plasma proteomic profile changes of heat-stressed dairy cows (Min et al., 2016). Moyes et al. (2013) used isobaric tags for relative and absolute quantitation based quantitative profiling of cow liver tissue and found potential hepatic biomarkers for different degrees of physiological imbalance of dairy cows in early and mid-lactation. Using the same method, Fan et, al (Fan et al., 2017) identified differentially expressed proteins related to a metabolic disorder of subclinical hypocalcemia. Moreover, proteomic analysis using liver tissues (Xu and Wang, 2008) and adipose tissues (Xu et al., 2019) has also been used in cow ketosis studies, which would provide novel opportunities to unravel the complex biology of the disease.

Recent advances in multi-omics approaches have significantly facilitated studies on the underlying mechanisms of complex metabolic diseases such as obesity or diabetes in humans (Oberbach et al., 2011). The combination of metabolomics and proteomics is often preferred as a powerful tool for exploring the network of interactions and regulatory events in diverse biological systems. We hypothesized that cows diagnosed with clinical ketosis would have altered metabolomic and proteomic profiles in comparison with the healthy controls. Therefore, a liquid chromatography-mass spectrometry (LC-MS) based metabolomics method and a Capillary-Flow Data-Independent Acquisition (DIA) LC-MS based proteomics method were used to get a comprehensive and system-wide understanding of ruminant ketosis. Through integration of metabolomic and proteomic data, we could identify the key regulators and build critical protein-metabolite networks responsible for the incidence and progression of ketosis in dairy cows.

## Materials and Methods

### Experimental Animals and Blood Serum Samples

All experimental procedures involved in this study were approved by the Institutional Animal Care and Use Committee of Sichuan Agricultural University (DKY-B20171906). The current study is a continuation of previous research, where the differentially expressed genes and pathways associated with ketosis were investigated using these animals (Wu et al., 2020). The animals and experimental design were fully described in the original article. In brief, a total of 74 multiparous Holstein cows at third parity with similar age, body condition score, and due dates (Supplementary Table 1) were enrolled at 3 weeks before parturition and raised in the same environment. All animals were fed regularly three times a day at 07:00, 13:00, and 19:00 with a total mixed ration, and the basal formulation may be found in Supplementary Table 2. Feed and water were offered *ad libitum*. Animals were managed by staff trained to identify medical problems and ketosis. Ketosis was determined at both prepartum (2 weeks before parturition) and postpartum (2 weeks after parturition) by testing plasma β-hydroxybutyrate (BHBA). A clinical ketosis case (case) was determined as a cow having BHBA concentration of ≥ 2.60 mM and a healthy control (control) with < 1.0 mM, respectively. Animals were excluded from the herd if diagnosed with clinical ketosis at prepartum or had other diseases during the whole experiment period. Among these cows, a subset of 19 animals were enrolled, with nine ultimately developing into clinical ketosis and 10 remaining healthy controls at postpartum.

Blood samples (10 mL) at both prepartum and postpartum were collected from coccygeal veins using vacutainer tubes before the morning feeding. After 30 min at room temperature for clot formation, samples were centrifuged at 3000 × *g* for 4°C for 15 min to obtain the corresponding blood serum sample. Serums were then stored in liquid nitrogen until used for further analysis. For these 19 finally enrolled animals, a total of 38 serum samples from two time points of both postpartum and prepartum were obtained and then divided into four groups: cases at postpartum (CK; BHBA = 2.79 ± 0.12 mM; *n* = 9), cases at prepartum (PCK; BHBA = 0.36 ± 0.05 mM; *n* = 9), controls at postpartum (HC; BHBA = 0.65 ± 0.22 mM; *n* = 10) and controls at prepartum (PHC; BHBA = 0.42 ± 0.08 mM; *n* = 10). For each group, all the serum samples were used for metabolomic analysis and five randomly selected samples were further subjected to proteomic analysis.

### Liquid Chromatography Mass Spectrometry Metabolomics Analysis

The LC-MS based metabolomic analysis has been described previously (Dunn et al., 2011; Luo et al., 2019). In brief, all 38 serum samples were slowly thawed at 4°C, and 100 μl of serum was mixed with 400 μl pre-cooled methanol. The mixed liquor was centrifugated at 12,000 rpm and 4°C for 10 min, and the supernatant was collected and blow-dried by vacuum concentration. Subsequently, the dried samples were dissolved with 150 μl of 2-chlorobenzylamine (4 ppm) methanol aqueous solution (4:1, 4°C). Finally, the supernatant was filtered through a 0.22 μm membrane and the prepared sample extracts were obtained for LC-MS analysis. For monitoring deviations of the analytical results and system stability over the entire experiment, 20 μl from each prepared sample were extracted and mixed for the preparation of quality control (QC) samples.

The liquid chromatographic separation was performed on a Thermo Ultimate 3000 system (Thermo Fisher Scientific Inc., Waltham, MA, United States) equipped with a Waters ACQUITY UPLC^®^ HSS T3 column (150 × 2.1 mm, 1.8 μm). The flow rate was 0.25 mL min^–1^ and the column temperature was maintained at 40°C. The mobile phase consisted of 0.1% formic acid in water (A) and 0.1% formic acid in acetonitrile (B) or 5 mM ammonium formate in water (C) and acetonitrile (D). Injection of 2 μl of each sample was done after equilibration. An increasing linear gradient of solvent B (v/v) was used as follows: 0∼1 min, 2% B/D; 1∼9 min, 2%∼50% B/D; 9∼12 min, 50%∼98% B/D; 12∼13.5 min, 98% B/D; 13.5∼14 min, 98%∼2% B/D; 14∼17 min, 2%B/D. In addition, the QC sample was used to optimize the liquid chromatographic separation condition, as it contained the most information of the whole serum samples. The MS experiment was executed on the Thermo Q Exactive Focus mass spectrometer (Thermo Fisher Scientific Inc., Waltham, MA, United States) with the spray voltage of 3.8 kV and −2.5 kV in positive ion mode (ESI^+^) and negative ion mode (ESI^–^), respectively. Data-dependent acquisition (DDA) MS/MS experiments were performed with HCD scan, and the normalized collision energy was 30 eV. Dynamic exclusion was implemented to remove some unnecessary information in MS/MS spectra.

### Data-Independent Acquisition Liquid Chromatography Mass Spectrometry Proteomics Analysis

The DIA large-scale proteomic method was described in detail previously (Bruderer et al., 2019). Briefly, all 20 serum samples were slowly thawed at 4°C, and then mixed with ammonium bicarbonate solution. Samples were reduced at 37°C for 1 h followed by alkylation in the dark for 1 h. Then, 100 μg of denatured serum were mixed with the ammonium bicarbonate buffer and 2.5 μg of trypsin and digested for 16 h at 37°C. Thereafter, the protein extracts were lyophilizated by freeze dryer according to the manufacture’s protocol. Desalting was performed using Sep-Pak C18 1CC Vac Cartridge (Waters, Milford, MA) following the manufacturer’s instructions. For library generation, pooled samples were fractionated using high pH reversed phase fraction chromatography (HPRP). 150 μl of digest was adjusted to pH 10 using pure ammonium formate, and then fractionated using HPRP separation on a H-Class UHPLC (Waters, Milford, MA) with a 2.1 × 150 mm BEH C18 1.7 μm column (Waters). Twelve fractions were collected and each fraction was dried in a vacuum concentrator for the next step. The fractions were resuspended with 40 μl solvent C containing 1× iRT kit, separated by nanoLC, and analyzed by on-line electrospray tandem mass spectrometry. Conditions for DDA analysis and DIA analysis were similar to those reported in Roland et al. (Bruderer et al., 2019).

The acquired MS spectra were analyzed by Mascot search engine (v.2.3.2; Matrix Science, London, United Kingdom) for protein identification by searching against the Bovine databases obtained from Uniprot^1^. DTA files were generated from the raw data files and then converted to Mascot generic files using Proteome Discoverer software (v.1.4.0.288). Trypsin was specified as the proteolytic enzyme and two missed cleavage was allowed. Carbamidomethyl of cysteine was used as a fixed modification, methionine oxidation as a variable modification. The initial peptide mass tolerance was set at 10 ppm in the first search and 5 ppm in the main search, and fragment (MS/MS) mass deviation was set to 20 ppm; false discovery rate (FDR) for peptide and protein identification of all searches were less than 5%. Each protein identification involved at least one unique peptide. For protein quantification, a protein had to contain at least one unique spectra. The quantitative protein ratios were weighted and normalized by the median ratio in Mascot^2^.

### Data Processing and Statistics

In the metabolomics analysis, all LC-MS data were extracted by ProteoWizard (v.3.0.878) and converted to mzXML format. All mass spectra were processed with peaks identification, peaks filtration, and alignment using the R package XCMS (Smith et al., 2006). The chromatographic peak data were normalized uniformly, and the multidimensional data were analyzed using SIMCA-P software (v.14.1). The metabolic peaks with relative standard deviations (RSDs) larger than 30% in QC samples were removed from the dataset (Dunn et al., 2011). Principal component analysis (PCA) was carried out to determine the global clustering and separation trends or possible outliers in an un-supervised manner. Orthogonal partial least squares discriminant analysis (OPLS-DA), a supervised model, was performed to obtain an overview of the complete data set and discriminate the inter-group differences. The model quality could be evaluated based on interpretation of variation for the X matrix (R^2^Y) and forecast ability of the model (Q^2^), which was discussed elsewhere (Yin et al., 2009). Generally, the model is believed to be reliable when *Q*^2^ > 0.4. The differentially accumulated metabolites (DAMs) were screened out using variable importance projection threshold (VIP > 1.0) in the OPLS-DA model and *p*-value in student’s *t*-test (*p* < 0.05) (Tian et al., 2015). Identification of metabolites was carried out by searching the reference standard MS/MS spectral library or the HumanMetabolome Database (HMDB^3^), Metlin^4^, or mzcloud^5^ database. The functional enrichment analysis of DAMs was performed based on the Kyoto Encyclopedia of Genes and Genomes (KEGG) database using MetaboAnalyst 4.0 online tool (Chong et al., 2018).

In the proteomics analysis, Student’s *t*-test was used to compare protein differences between groups and to calculate *p*-values. Protein with a fold change of 1.5 and *p* < 0.05 was considered as differentially abundant protein (DAP) based on the published reference (Guo et al., 2019). We used PCA and partial least squares discriminant analysis (PLS-DA) to visualize the distribution of the samples between case and control groups and detect potential outliers. Functional enrichment analysis was performed using the differentially expressed proteins between different groups by functional categorization of Gene Ontology (GO) terms with agriGO toolkit (Tian et al., 2017), and KEGG pathway analysis was performed by KOBAS 3.0 (Xie et al., 2011).

Pearson correlation analysis was conducted for evaluating the metabolomics and proteomics integration. For this, the expression data of both DAMs and DAPs related to ketosis were calculated. Then, only the protein and metabolite with high correction (|*R*| ≥ 0.7) and *p*-value < 0.05 were considered. Finally, pathway over-representation analysis was conducted using Integrated Molecular Pathway Level Analysis (IMPaLA) (Kamburov et al., 2011).

## Results

### Quality Assessment of LS-MS Data and Metabolites Identification

**FIGURE 1:**
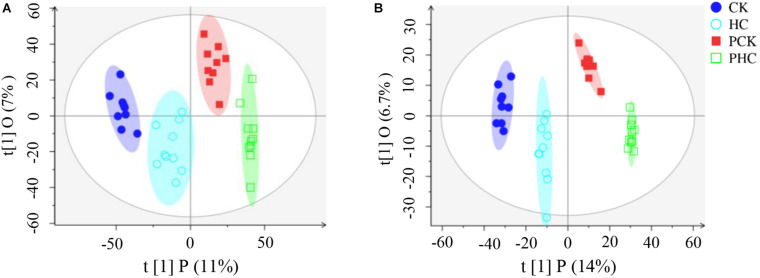
Orthogonal partial least squares discriminant analysis (OPLS-DA) score scatter plots of all grouped sera in the study. **(A)** All the four groups in ESI^+^ mode (*R*^2^*X* = 0.18, *R*^2^*Y* = 0.976, *Q*^2^ = 0.826), and **(B)** and ESI^–^ mode (*R*^2^*X* = 0.25, *R*^2^*Y* = 0.993, *Q*^2^ = 0.848), respectively.

The pooled QC sample was applied to ensure the reproducibility of the LC-MS system. The overlapped total ion chromatograms (TIC) of QC samples in positive and negative ion modes demonstrated the strong repeatability of the instruments, and more than 70% of main peaks had RSDs lower than 30% (Supplementary Figure 1). These results represented the robustness of the system. Therefore, the method was deemed acceptable for our subsequent metabolic analysis. Multivariate analysis of OPLS-DA was used to detect potential outliers and identify features potentially responsible for causing the variation between different groups of sera. Generally, the model was jointly assessed using R^2^ (model fit) and Q^2^ (predictive power) and the model is believed to be reliable when the *R*^2^*Y* and *Q*^2^ values > 0.4. As shown in Figure 1, the OPLS-DA score plot could separately distinguish each of the four sera groups, indicating the differential metabolomic profiling of the four groups. Furthermore, in the positive ion mode, *R*^2^*X* = 0.297, *R*^2^*Y* = 0.999, and *Q*^2^ = 0.936 (Figure 1A), whereas in the negative ion mode, *R^2^X* = 0.25, *R*^2^*Y* = 0.993, and *Q*^2^ = 0.848 (Figure 1B). Both the *R*^2^*Y* and *Q*^2^ values of the models were greater than 0.4, indicating that the models were predictable and reliable to discriminate among the four groups. After rigorous quality control and identification, we obtained 97 metabolites (Supplementary Table 3) among all samples.

### Comparisons of the Metabolomic Profiles of Sera From Different Groups

**FIGURE 2:**
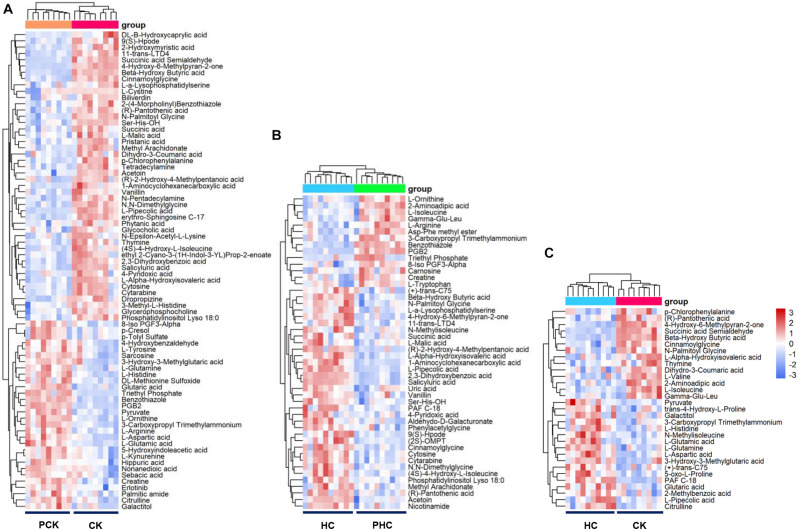
Heatmap of the differentially accumulated metabolites (DAMs) in sera of different comparisons. **(A)** Hierarchical clustering analysis of the 76 DAMs between CK vs. PCK, and **(B)** the 48 DAMs between HC vs. PHC, and **(C)** the 31 DAMs between CK vs. HC, respectively.

We next examined the metabolomic profiles of sera from different groups. Firstly, we examined the variations in metabolomic profiles of cases during parturition. Multivariate analysis showed that when sera metabolites from CK were compared to those from PCK, both score plots from the PCA (Supplementary Figure 2) and OPLS-DA (Supplementary Figure 3) exhibited a clear separation without any overlap. It is noted that both the *R*^2^*Y* and *Q*^2^ values of OPLS-DA models from positive ion mode and negative ion mode were greater than 0.4 (Supplementary Figure 3), demonstrating that the models were stable and reliable. We next examined dissimilarities in the abundance of identified metabolites between these two groups. Based on the VIP value in the OPLS-DA model > 1 and *p*-value in student’s *t*-test < 0.05, a total of 76 DAMs were identified. Of these, the level of 46 metabolites had increased, whereas those of 30 had decreased in CK group with respect to the level of PCK group (Supplementary Table 4). Sera belonging to a group (CK or PCK) clustered together perfectly (Figure 2A).

Secondly, in order to reveal the successful adaption changes in global metabolites during the transition phase, metabolomic profiles of controls during parturition were analyzed using multivariate analysis. Our results of PCA (Supplementary Figure 2) and OPLS-DA (Supplementary Figure 3) showed that sera metabolites from HC and those from PHC were clearly separated into two parts. We also observed that both the *R*^2^*Y* and *Q*^2^ values of OPLS-DA models from both positive ion mode and negative ion mode were greater than 0.4 (Supplementary Figure 3), indicating a satisfactory effectiveness of the models which can be used to identify the difference between two groups. Based on the criteria of VIP > 1 and *p* < 0.05, 48 variables were screened out with 34 metabolites upregulated and 14 downregulated ones in the HC group when compared with PHC group (Supplementary Table 5). The hierarchical clustering illustrated that these metabolites clearly segregated the samples into two groups (Figure 2B).

Thirdly, we used PCA and OPLS-DA score plots to detect potential outliers and identify features potentially responsible for causing the variation between cases and controls at postpartum. Our results of PCA (Supplementary Figure 2) and OPLS-DA (Supplementary Figure 3) also showed excellent separation between CK and HC. The parameters of the OPLS-DA models from positive ion mode and negative ion mode showed that both the *R*^2^*Y* and *Q*^2^ values were greater than 0.4 (Supplementary Figure 3). This indicated that these are reliable and predictable models to discriminate between the two groups. To identify the DAMs between the CK and HC groups, we compared the abundance of identified metabolites between these two groups. A total of 31 DAMs were obtained from the comparison, 14 of which had a higher relative abundance in the CK than HC group; the other 17 metabolites significantly decreased in the CK group (Supplementary Table 6). Cluster hierarchization showed that the clusters of these two groups were obviously separated (Figure 2C).

**FIGURE 3:**
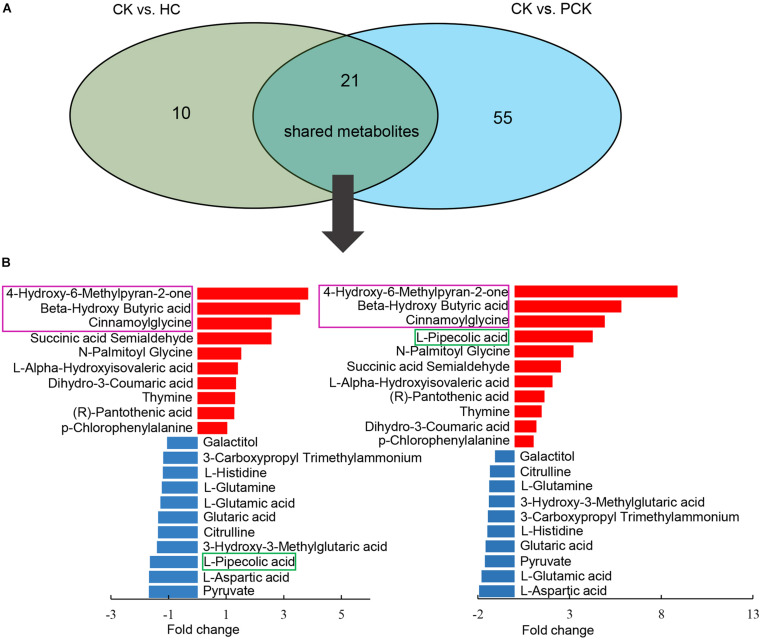
Comparison of metabolite profiling between CK vs. HC and CK vs. PCK. **(A)** 21 shared metabolites from these two comparisons were identified and which showed similar up- or down- accumulated pattern but L-Pipecolic acid. **(B)** Bar plot (left) showed the fold change of shared metabolites in CK vs. HC and (right) CK vs. PCK, of which, red represents high and blue represents low in CK group.

In addition, we found 21 shared metabolites between the comparisons of CK vs. HC and CK vs. PCK (Figure 3A). Strikingly, of these shared metabolites, 10 were consistently upregulated and the other 10 consistently downregulated in the CK group; only one metabolite of L-Pipecolic acid did not show agreement in the direction of the fold change between comparisons of CK vs. HC and CK vs. PCK (Figure 3B), indicating that these 20 metabolites were consistent with the clinical determination of the cases. The most upregulated metabolites in the CK group included the metabolites of 4-Hydroxy-6-Methylpyran-2-one, BHBA, and cinnamoylglycine (Figure 3).

### Functional Implications of Differentially Accumulated Metabolites

**FIGURE 4:**
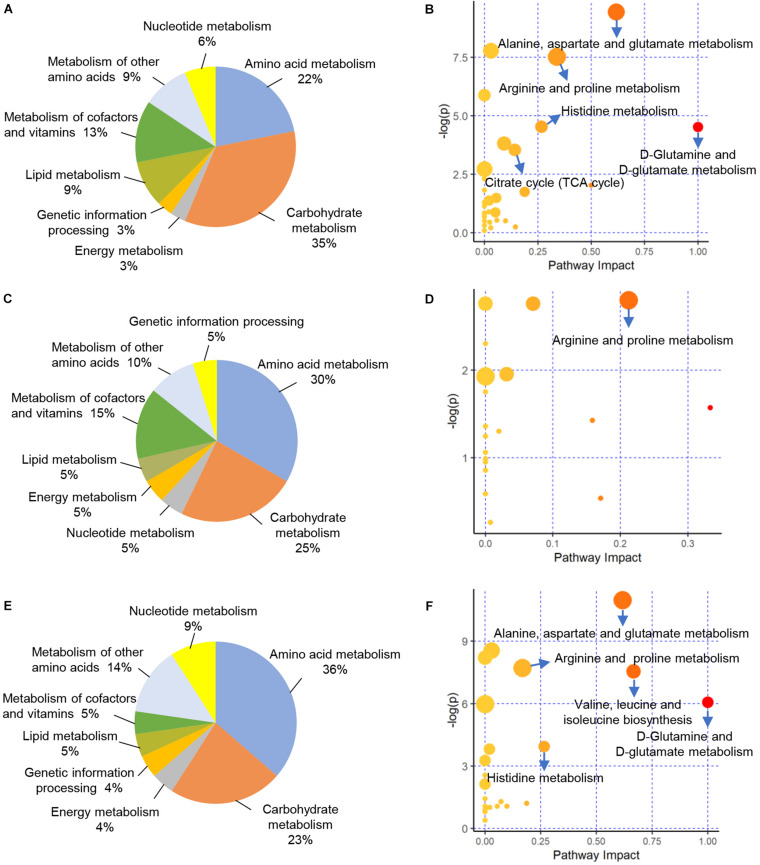
Functional classification and enrichment analysis of differentially accumulated metabolites (DAMs) from different comparisons. **(A,C,E)** Functional classification of these DAMs identified between CK vs. PCK, HC vs. PHC, and CK vs. HC, respectively. **(B,D,F)** Enrichment results of these DAMs identified between CK vs. PCK, HC vs. PHC, and CK vs. HC, respectively.

We evaluated the interactions of the 76 DAMs identified between CK vs. PCK, which would help to unravel potential metabolic changes contributed to ketosis from prepartum to postpartum. The enrichment analysis revealed that these DAMs were enriched in 32 KEGG pathways. Among these pathways, carbohydrate metabolism (35%), amino acid metabolism (22%), and metabolism of cofactors and vitamins (13%) accounted for a large proportion (Figure 4A). Furthermore, a total of five pathways of “D-Glutamine and D-glutamate metabolism”, “Alanine, aspartate, and glutamate metabolism”, “Arginine and proline metabolism”, “Histidine metabolism”, and “Citrate cycle (TCA cycle)” were the most enriched pathways (*p* < 0.05, impact value > 0.10) (Figure 4B). For the 48 DAMs identified between HC vs. PHC, these would help to unravel the metabolic pathways involved in successful adaption from prepartum to postpartum. The enrichment analysis revealed that these DAMs were enriched in 20 KEGG pathways. Among these pathways, amino acid metabolism (30%), carbohydrate metabolism (25%), and metabolism of cofactors and vitamins (15%) accounted for a large proportion (Figure 4C). The functional impact pathways were shown in Figure 4D, by which we found only the pathway of “Arginine and proline metabolism” was significantly changed in lactating cows.

To further investigate metabolic variations of cases and controls at postpartum, we mapped the 31 DAMs identified between CK vs. HC to the KEGG database. The results of enrichment demonstrated that there were 22 pathways enriched. Among these pathways, amino acid metabolism (36%), carbohydrate metabolism (23%), and metabolism of cofactors and vitamins (14%) accounted for a large proportion (Figure 4E). The five significantly enriched pathways included “D-Glutamine and D-glutamate metabolism”, “Valine, leucine, and isoleucine biosynthesis”, “Alanine, aspartate, and glutamate metabolism”, “Histidine metabolism”, and “Arginine and proline metabolism” (Figure 4F).

### Protein Identification and Multivariate Analysis

**FIGURE 5:**
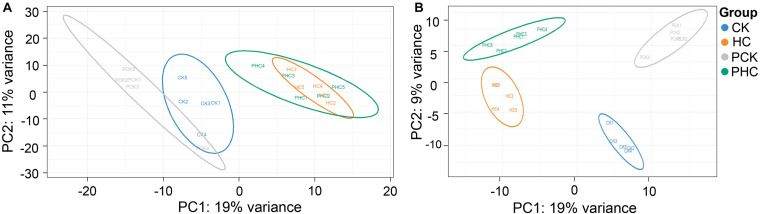
Multivariate analysis of proteins in sera from different groups. **(A)** Principal component analysis (PCA) score scatter plots of all the four grouped sera. **(B)** Partial least squares discriminant analysis (PLS-DA) score scatter plots of all the four grouped sera.

To further understand the systematic changes associated with metabolic adaptions to transition phase stress as well as metabolic variations associated with ketosis during the transition period, the proteomic profiles among cases and controls at both prepartum and postpartum were carried out. Of the total sequenced spectra, 7,036 were mapped to the bovine reference protein database. Of these, 4,072 were uniquely mapped to specific peptides. In total, 540 proteins were identified under the 5% false discovery rate threshold at both the peptide and protein levels. Next, all the 540 proteins were subjected to PCA analysis for clustering all the samples. The first two principle components accounted for 30% of total variance (Figure 5A). This panel did not show a clear separation between sera from HC and PHC groups. However, it can be easily observed that sera within each group tended to cluster together (Figure 5A). Subsequently, the PLS-DA model was used for further multivariate analysis, which revealed that the proteomics of each group could be clearly distinguished from the others (Figure 5B), indicating the differential proteomic profiling of the four groups of sera. In summary, clear separation in proteomic profiles was found among sera from cases and controls.

### Comparisons of the Proteomic Profiles of Sera From Different Groups

**FIGURE 6:**
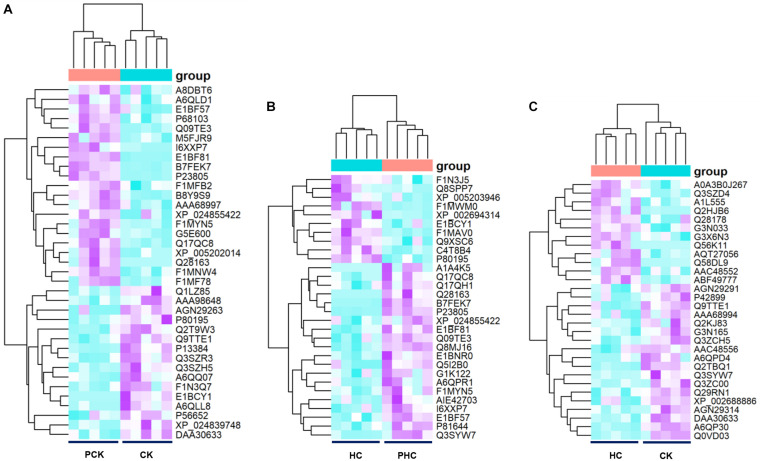
Heatmap of the differentially abundant proteins (DAPs) between sera from different comparisons. **(A)** Hierarchical clustering analysis of the 37 DAPs between CK vs. PCK, and **(B)** 30 DAPs between HC vs. PHC, and **(C)** 30 DAPs between CK vs. HC, respectively.

To survey the DAPs associated with transition phase stress as well as ketosis, we compared the abundance of identified proteins among the following three comparisons. Firstly, we identified 37 DAPs in sera between the CK vs. PCK (fold change > 1.5 and *p* < 0.05), of which 16 were upregulated and 21 were downregulated in the CK group (Supplementary Table 7). A heatmap of these DAPs was generated to visualize expression patterns across all 10 sera samples, and expression patterns in the heatmap were accompanied by hierarchical clustering of proteins (horizontal axis) and samples (vertical axis) (Figure 6A). Secondly, we identified 30 DAPs, with 10 significantly higher and 20 significantly lower relative concentration proteins in the HC group when compared with PHC group (Supplementary Table 8). Cluster hierarchization using the expression data of these 30 DAPs confirmed the presence of two distinct groups (Figure 6B). Thirdly, a total of 30 DAPs was identified between CK vs. HC, with 18 upregulated and 12 downregulated in CK group compared with HC group (Supplementary Table 9). The protein abundance data of these 30 DAPs revealed an obvious separation of two parts (Figure 6C), which confirmed the presence of discriminating features between CK and HC groups.

### Functional Enrichment Analysis of Differentially Abundant Proteins

**FIGURE 7:**
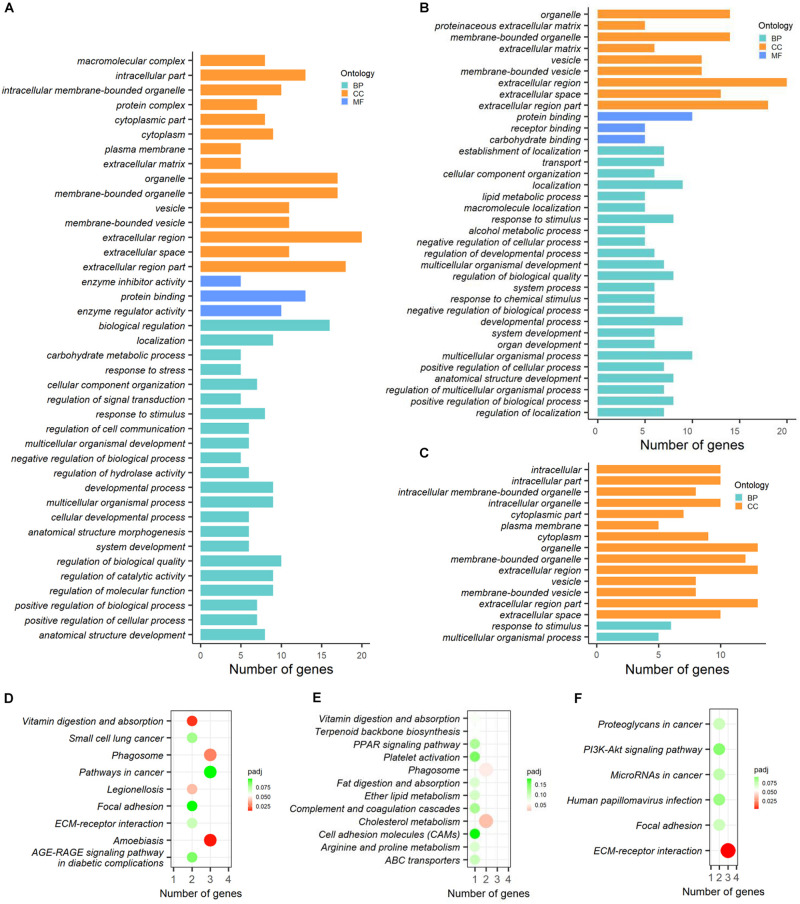
Functional enrichment analysis of differentially abundant proteins (DAPs) between different comparisons. **(A–C)** GO categories enriched by DAPs between CK vs. PCK, HC vs. PHC, and CK vs. HC, respectively. **(D–F)** KEGG pathways enriched by DAPs between CK vs. PCK, HC vs. PHC, and CK vs. HC, respectively.

The functional enrichment analysis of DAPs based on GO categories was performed and the significantly (*p*-adjusted < 0.05) enriched terms were shown in Figures 7A–C. By which, the 37 DAPs obtained between CK vs. PCK were enriched in 40 GO terms. Three of these terms corresponded to molecular function (MF), namely protein binding, enzyme regulator activity, and enzyme inhibitor activity, and most of these proteins were concentrated in protein binding (Figure 7A). For biological process (BP) ontology, 22 terms were enriched, and most of these proteins were enriched in the terms of biological regulation, regulation of biological quality, and regulation of molecular function (Figure 7A). The cellular component (CC) ontology presented 15 enriched terms, and those of extracellular region, extracellular region part, membrane-bounded organelle, and extracellular region were ranked at the top of the category (Figure 7A). In addition, a total of 36 GO terms were obtained by DAPs between HC vs. PHC. For biological processes, most proteins were enriched in multicellular organismal process, localization, developmental process, and response to stimulus (Figure 7B). For molecular function, the top term was protein binding (Figure 7B), while no MF terms were enriched by DAPs between CK vs. HC (Figure 7C). Meanwhile, the proteins participate in several CC terms, such as extracellular region, extracellular region part, organelle, and membrane-bounded organelle, and these terms had high ratios among the DAPs that were identified among all three comparisons within each comparison.

For cellular components, most proteins were enriched in the extracellular region, extracellular region part, and membrane-bounded organelle. For molecular function, the top term was protein binding (Figure 7B), while no MF terms were enriched by DAPs between CK vs. HC (Figure 7C).

The KEGG analysis of DAPs allowed us to better understand the key proteins and pathways affected by transition phase stress and ketosis. By which, the KEGG pathways enriched by DAPs between different comparisons were displayed in Figures 7D–F. A total of four pathways, including amebiasis, vitamin digestion and absorption, phagosome, and legionellosis, were significantly (*p*-adjusted < 0.05) enriched by DAPs between CK vs. PCK (Figure 7D); those between HC vs. PHC were cholesterol metabolism and phagosome (Figure 7E), and those between CK vs. HC were ECM-receptor interaction (Figure 7F).

### Integrating Metabolomics and Proteomics Pathway Analysis

**FIGURE 8:**
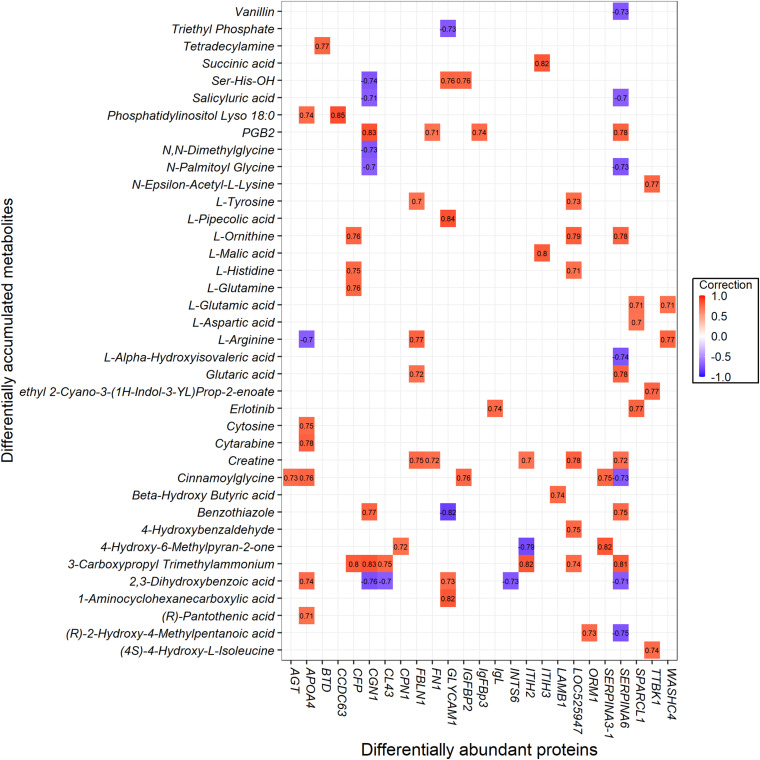
The interaction value between the differentially abundant proteins (DAPs) and differentially accumulated metabolites (DAMs).

To investigate the protein and metabolite regulatory network of ketosis implicated in transition cows, we performed a pathway over-representation analysis using IMPaLA tool. By which, the DAMs and DAPs obtained from both comparisons of CK vs. PCK and CK vs. HC were used to identify significantly perturbed pathways. In total, 85 metabolites and 53 annotated proteins were subjected to Pearson correlation analysis (Supplementary Table 10). The results showed that 38 metabolites had 78 strong corrections (*R* ≥ 0.7) with 25 proteins (Figure 8).

Based on the results, interaction networks between the 38 metabolites and 25 proteins were organized. The list of pathways with joint *p*-values < 0.05 is shown in Supplementary Table 11. Ketosis altered a wide range of metabolic pathways, among them mainly metabolism, metabolism of proteins, and metabolism of angiotensinogen to angiotensins. The cellular processes involved were ones such as gene expression (transcription), RNA polymerase II transcription, and post-translational protein modification. Also involved were vitamin metabolism (e.g., vitamin digestion and absorption, metabolism of vitamins and cofactors, metabolism of water-soluble vitamins and cofactors) and signaling (e.g., G alpha (q) signaling events, GPCR ligand binding, GPCR downstream signaling, signaling by GPCR, and signal transduction). Other pathways associated with the vascular system (hemostasis, ion channels, and their functional role in vascular endothelium) and disease (amebiasis) were identified.

## Discussion

Because blood reflects the physiological and pathological states in body, the blood-based profiles would be a powerful means in all kinds of biological studies (Mohr and Liew, 2007). For ruminants, some blood parameters, such as glucose, fructosamine, insulin, metabolites, enzymes, and proteins, may indicate their nutrient status (Stengärde et al., 2008; Puppel and Kuczynska, 2016). Although several biomarkers and the pathogenesis of ketosis have been recently proposed, the understanding of serum metabolomic and proteomic changes during the incidence and progression of ketosis remains limited (Abuajamieh et al., 2016; Li et al., 2018; Wang et al., 2018). However, these studies focused exclusively on samples collected from sick cows and matched controls without taking prospective samples into account. Here, we used high-throughput metabolomic and proteomic analysis on serum collected at both prepartum and postpartum to identify differentially expressed metabolites and proteins between cases and controls and explored their functions involved in ketosis. In addition, we extended our investigations to uncover serum metabolomic and proteomic changes from prepartum to postpartum, which could provide potentially new insights to the metabolic changes to adapt transition phase stress.

As an effort to elucidate the metabolic changes to adapt transition phase stress, a comparison of metabolomics and proteomics between HC vs. PHC were performed, and 48 metabolites (Figure 2B) and 30 proteins (Figure 6B) were found to be differentially expressed. These metabolites were found to be significantly enriched in arginine and proline metabolism, while these proteins were mainly enriched into biological processes and pathways associated with cholesterol metabolism and phagosome. This finding is consistent with a previous study that showed that amino acid metabolism and energy metabolism are related changes in transition cows (Luo et al., 2019). Nevertheless, it should be noted that we did not demonstrate the inflammation-related pathways as we have previously shown from transcriptome data with the same animals. A possible reason for this observation is that there is not always a relationship between gene expression and protein expression.

Multivariate analysis revealed significant differences in both the metabolomic and proteomic profiles between cases and controls, revealing an evident impact of ketosis on serum metabolites and proteins. Our results demonstrated that those metabolites changed between cases and controls at postpartum were significantly enriched into the pathways related to amino acid metabolism, carbohydrate metabolism, nucleotide metabolism, and amino acid biosynthesis and metabolism. In the meantime, functional analysis showed that metabolites changed between cases at prepartum and postpartum were also enriched in the same pathways. Thus, the results altogether suggested that the landscape of sera metabolites may direct the dynamic changes in level of compounds involved in particular pathways during the incidence and progression of ketosis. These pathways identified at a metabolomic level will ultimately improve our understanding of ketosis. At a proteomic level, KEGG pathway analysis indicated that these proteins were involved in disease-related pathways, such as amebiasis, vitamin digestion and absorption, phagosome, legionellosis, and ECM-receptor interaction; part of these results are in accordance with our previous work based on transcriptomic analysis (Wu et al., 2020) and work from others (Xu and Wang, 2008; Trevisi and Minuti, 2018). It was difficult to uncover molecular mechanisms for ketosis during the transition period although the level of each compound and the abundance of each protein could be determined. We used IMPaLA to analyze the high correlated metabolites and proteins for integrating pathway analysis. It revealed connections of ketosis related metabolites and proteins, which were significantly enriched in a wide range of metabolic pathways, cellular processes, vitamin metabolism, and signaling. Of note, most of these pathways have been shown to have essential roles in the regulation of ketosis (Sun et al., 2014; Wang et al., 2016; Shahzad et al., 2019).

Negative energy balance is the pathological basis of ketosis. It was reported that elevated ketone bodies, such as BHBA, acetone, and acetoacetate, could serve as the metabolic biomarkers for detecting ketosis (Enjalbert et al., 2001). Additionally, concentrations of NEFA in blood is also used as an indicator of negative energy balance in dairy cows (Oetzel, 2004). Currently, the concentration of NEFA, BHBA, and glucose are commonly used as indicators of negative energy balance (Asl et al., 2011; Xia et al., 2012). It is well known that BHBA is the most common biomarker for evaluation and establishment of ketosis (Iwersen et al., 2009; González et al., 2011). In a previous study, several metabolites were identified to possibly predict or discriminate ketotic cows using a plasma targeted quantitative metabolomics approach (Hailemariam et al., 2014). In this study, the most consistently elevated metabolites in the CK group included 4-Hydroxy-6-Methylpyran-2-one, BHBA, and cinnamoylglycine. We proposed that 4-Hydroxy-6-Methylpyran-2-one and cinnamoylglycine could be potentially used as new alterative indicators to diagnose ketosis. Nevertheless, further studies are warranted to validate these results in large populations.

The limitation of this study is a relatively small sample size (nine cases and 10 controls) was conducted to explore the sera metabolomic and proteomic profiles. We applied the stringent inclusion criteria of 2.60 mmol/L plasma BHBA concentration for clinical ketosis, which could provide enhanced power to avoid false-positive of case animals. Even through proteomics costs less than before, it is still unaffordable to use on a large number of samples tested using DIA LC-MS based proteomics method. The statistical evaluation to determine differentially expressed proteins between groups was therefore limited. However, data from metabolomics and proteomics can provide complementary and inherent validation information with each other, and thus, integrating these two data sets can partially compensate for the relatively small sample sizes (Qiu et al., 2020).

## Conclusion

In summary, our results comprehensively revealed the metabolomic and proteomic profiles associated with the incidence and progression of ketosis in dairy cows during the transition period. The involved pathways have been successfully identified. Also, the metabolites of 4-Hydroxy-6-Methylpyran-2-one and cinnamoylglycine could be used as potential indicators to diagnose ketosis.

## Data Availability Statement

The raw data supporting the conclusions of this article will be made available by the authors, without undue reservation.

## Ethics Statement

The animal study was reviewed and approved by Institutional Animal Care and Use Committee of Sichuan Agricultural University (DKY-B20171906).

## Author Contributions

Z-LW and S-JL conceived and designed the experiments. Z-LW, S-YC, XJ, and JW performed the experiments. Z-LW and S-YC analyzed the data. Z-LW wrote the manuscript. S-YC, SH, and S-JL reviewed and edited the manuscript. All authors read and approved the final version of the manuscript.

## Conflict of Interest

The authors declare that the research was conducted in the absence of any commercial or financial relationships that could be construed as a potential conflict of interest.

## Footnotes

1 https://www.uniprot.org/news/2018/02/28/release

2 http://www.matrixscience.com/help/quant_statistics_help.html

3 http://www.hmdb.ca

4 http://metlin.scripps.edu

5 https://www.mzcloud.org

## Supplementary Material

Supplementary Figure 1 Principal component analysis (PCA) score scatter plots of sera from four groups (CK, HC, PCK, and PHC) and quality control (QC) based on sera LC-MS data. **(A)** PCA plot for metabolites obtained in ESI^+^ mode and **(B)** ESI^–^ mode.

Supplementary Figure 2 Principal component analysis (PCA) score scatter plots of sera show excellent separation between different comparisons. **(A,C,E)** PCA score scatter plots of metabolite profile between CK vs. PCK, HC vs. PHC, and CK vs. HC in ESI^+^ mode, and **(B,D,F)** those in ESI^–^ mode.

Supplementary Figure 3 Orthogonal partial least squares discriminant analysis (OPLS-DA) score scatter plots show excellent separation between different comparisons. **(A,C,E)** OPLS-DA score scatter plots of metabolite profile between CK vs. PCK (*R*^2^*X* = 0.297, *R*^2^*Y* = 0.999, *Q*^2^ = 0.936), HC vs. PHC (*R*^2^*X* = 0.242, *R^2^Y* = 0.987, *Q*^2^ = 0.814), and CK vs. HC (*R*^2^*X* = 0.19, *R*^2^*Y* = 0.995, *Q*^2^ = 0.716) in ESI^+^ mode, and **(B,D,F)** those between CK vs. PCK (*R^2^X* = 0.337, *R*^2^*Y* = 0.997, *Q*^2^ = 0.94), HC vs. PHC (*R*^2^*X* = 0.246, *R*^2^*Y* = 0.994, *Q*^2^ = 0.891), and CK vs. HC (*R*^2^*X* = 0.236, *R*^2^*Y* = 0.979, *Q*^2^ = 0.676) in ESI^–^ mode.
