# Supplementary material for: Metabolomic and Proteomic Profiles Associated With Ketosis in Dairy Cows
Source: Front Genet. 2020 Dec 16;11:551587. doi: 10.3389/fgene.2020.551587 (PMC7772412; doi:10.3389/fgene.2020.551587)
Supplement: Supplementary file 2 [file Table_2.DOCX]

**Table S2:** Ingredient and nutrient composition of the diets.

| **Item** | **parts** |
| --- | --- |
| **Diet component**  alfalfa grass | **Percentage composition**  15.45 |
| oat grass | 10.15 |
| corn silage | 17.15 |
| corn | 22.05 |
| wheat bran | 2.65 |
| soya bean meal | 8.80 |
| rapeseed meal | 1.30 |
| cottonseed meal | 1.30 |
| whole cottonseed | 11.00 |
| expand soybean | 4.40 |
| common salt | 0.30 |
| calcium hydrophosphate | 1.55 |
| microelement additive | 0.50 |
| magnesium oxide | 0.30 |
| baking soda | 1.80 |
| fatty acid calcium | 1.30 |
| **Nutrient component** | **Content (% DM basis)** |
| RUP | 31.82 |
| CF | 17.10 |
| NDF | 40.98 |
| ADF | 26.43 |
| Ca | 0.89 |
| P | 0.58 |
| Na | 0.64 |
| K | 1.34 |
| Mg | 0.40 |
| NEL, MJ/kg of DM | 6.10 MJ/Kg |

DM, dry matter; RUP, rumen undegradable protein; CF, crude protein; NDF, neutral detergent fiber; ADF, acid detergent fiber; Ca, calcium; P, phosphide; Na, sodium; K, potassium; Mg, magnesium.
