# Supplementary figures and images for: Metabolomic and Proteomic Profiles Associated With Ketosis in Dairy Cows
Source: Front Genet. 2020 Dec 16;11:551587. doi: 10.3389/fgene.2020.551587 (PMC7772412; doi:10.3389/fgene.2020.551587)

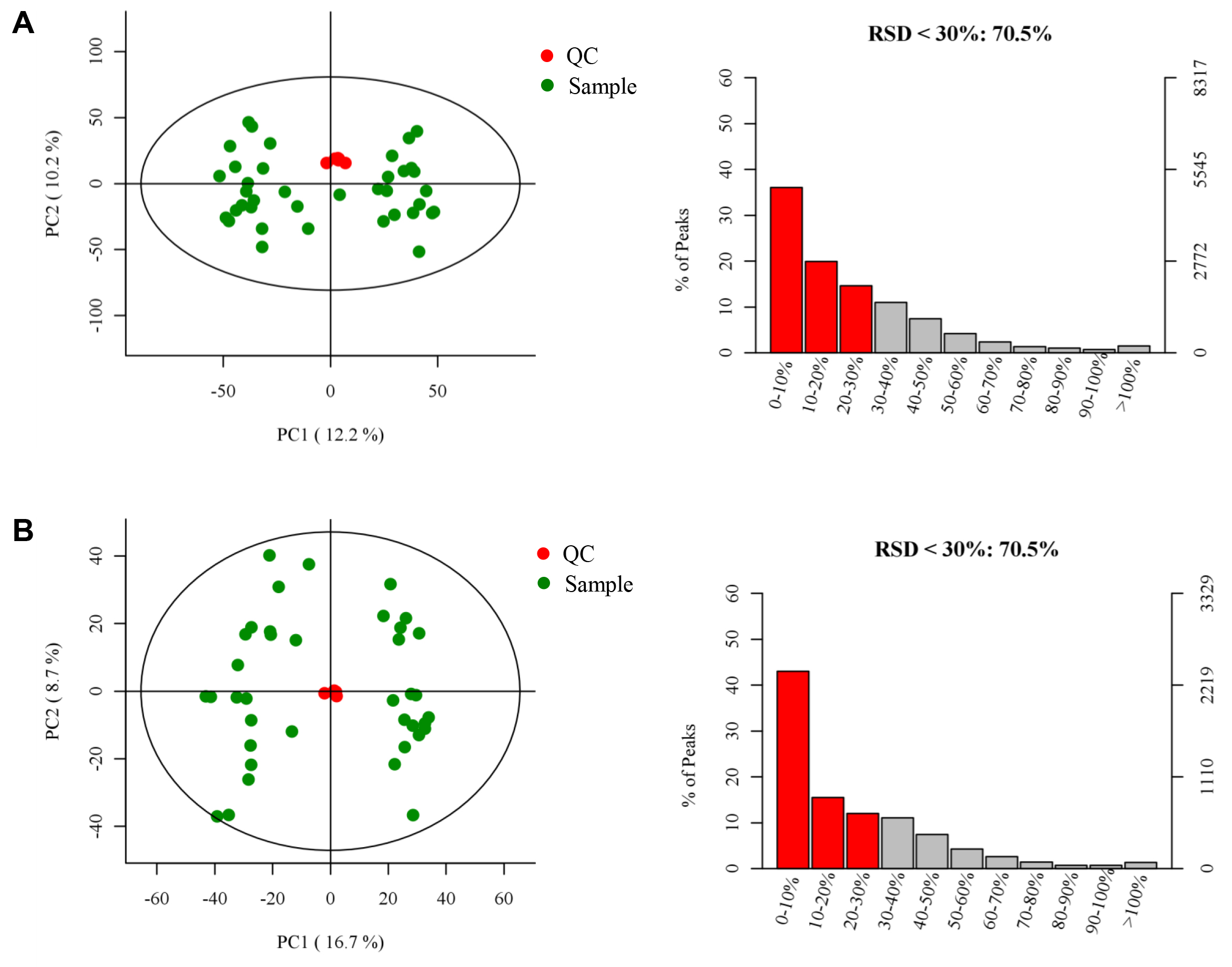

Supplement: Supplementary Figure 1 — Principal component analysis (PCA) score scatter plots of sera from four groups (CK, HC, PCK, and PHC) and quality control (QC) based on sera LC-MS data. (A) PCA plot for metabolites obtained in ESI+ mode and (B) ESI– mode. [file Image_1.JPEG]

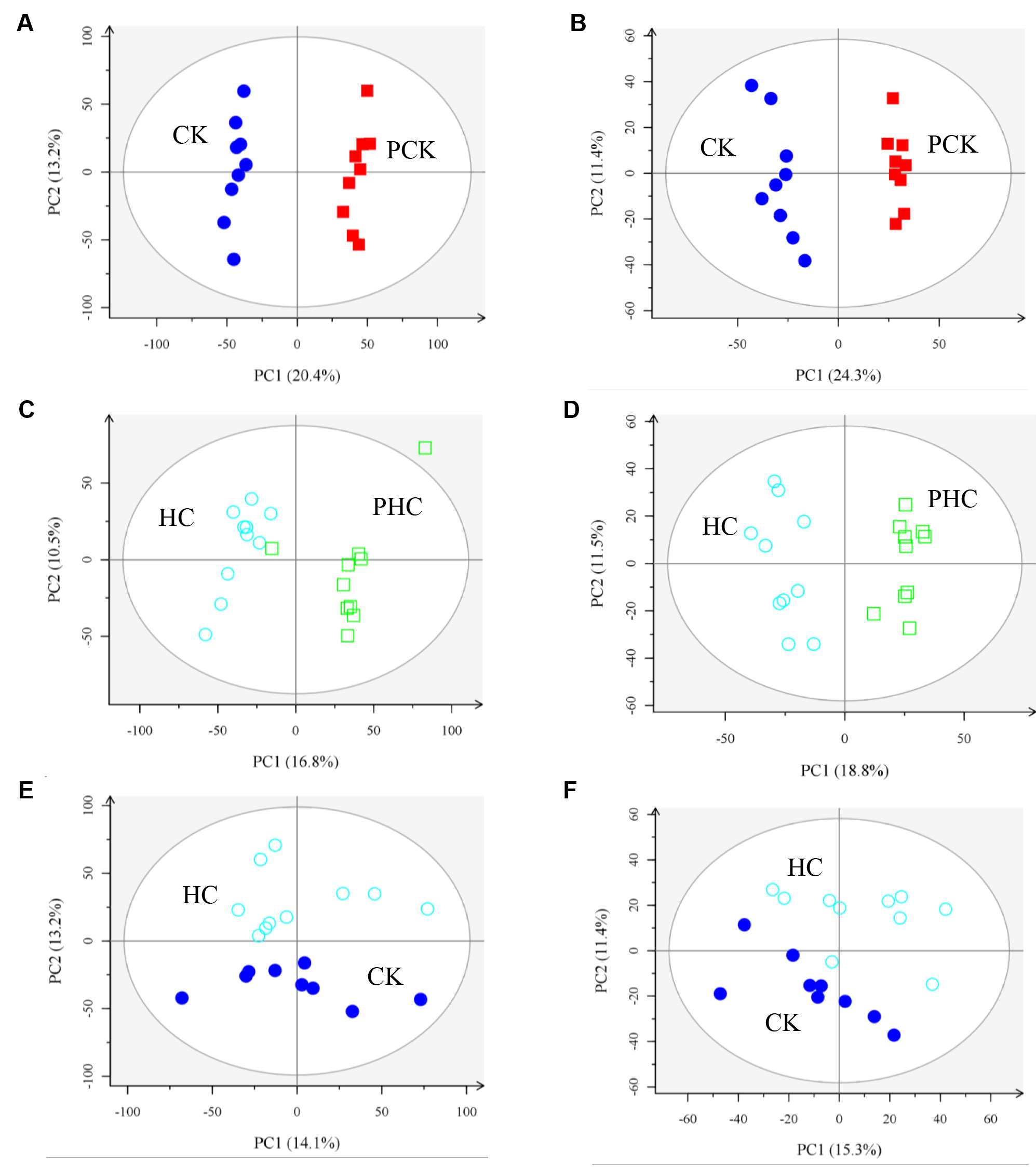

Supplement: Supplementary Figure 2 — Principal component analysis (PCA) score scatter plots of sera show excellent separation between different comparisons. (A,C,E) PCA score scatter plots of metabolite profile between CK vs. PCK, HC vs. PHC, and CK vs. HC in ESI+ mode, and (B,D,F) those in ESI– mode. [file Image_2.JPEG]

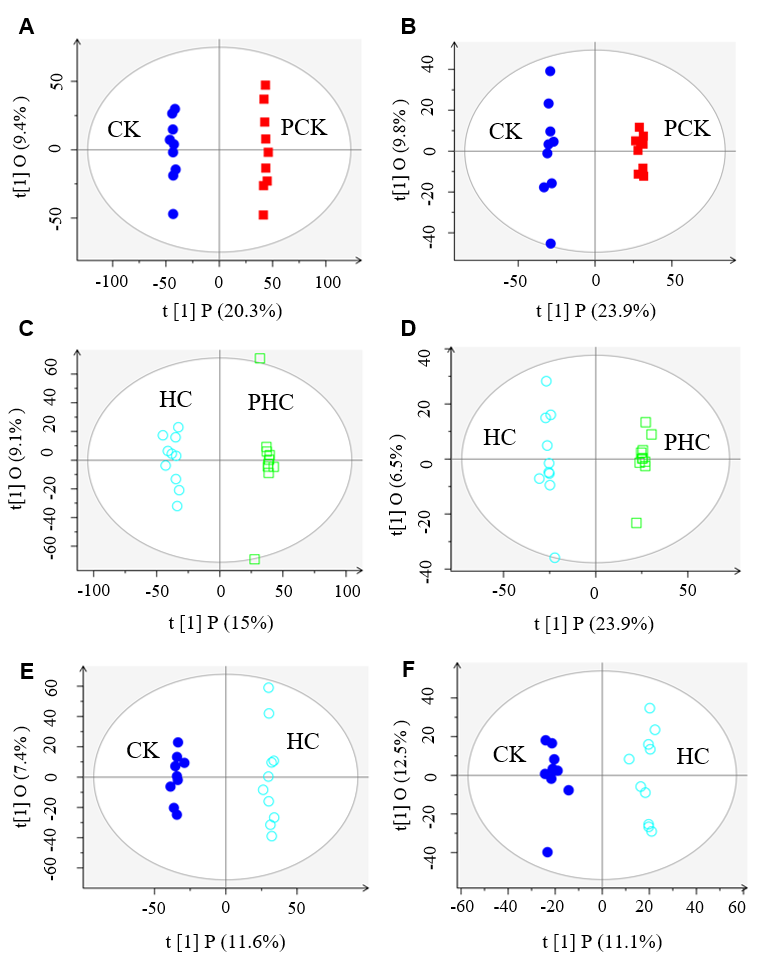

Supplement: Supplementary Figure 3 — Orthogonal partial least squares discriminant analysis (OPLS-DA) score scatter plots show excellent separation between different comparisons. (A,C,E) OPLS-DA score scatter plots of metabolite profile between CK vs. PCK (R2X = 0.297, R2Y = 0.999, Q2 = 0.936), HC vs. PHC (R2X = 0.242, R2Y = 0.987, Q2 = 0.814), and CK vs. HC (R2X = 0.19, R2Y = 0.995, Q2 = 0.716) in ESI+ mode, and (B,D,F) those between CK vs. PCK (R2X = 0.337, R2Y = 0.997, Q2 = 0.94), HC vs. PHC (R2X = 0.246, R2Y = 0.994, Q2 = 0.891), and CK vs. HC (R2X = 0.236, R2Y = 0.979, Q2 = 0.676) in ESI– mode. [file Image_3.PNG]
